# Effects of dietary oils prepared from the internal organs of the Japanese giant scallop (*Patinopecten yessoensis*) on cholesterol metabolism in obese type‐II diabetic KK‐*A^y^* mice

**DOI:** 10.1002/fsn3.1967

**Published:** 2020-10-26

**Authors:** Koki Sugimoto, Ryota Hosomi, Munehiro Yoshida, Kenji Fukunaga

**Affiliations:** ^1^ Laboratory of Food and Nutritional Sciences Faculty of Chemistry, Materials and Bioengineering Kansai University Suita Japan

**Keywords:** n-3 polyunsaturated fatty acid, eicosapentaenoic acid, docosahexaenoic acid, Japanese giant scallop (Patinopecten yessoensis), cholesterol metabolism

## Abstract

Our research team has successfully prepared high‐quality scallop oil (SCO), containing high eicosapentaenoic acid (EPA) and phospholipids (PL) from the internal organs of Japanese giant scallop (*Patinopecten yessoensis*) which is the largest unutilized marine resource in Japan. In this study, we prepared SCOs from scallop internal organs obtained from Mutsu (Aomori) and Uchiura (Hokkaido) bays in Japan, and named them SCO‐M and SCO‐U, respectively. This study aimed to investigate the effects of dietary SCO‐M and SCO‐U on cholesterol metabolism in obese type‐II diabetic KK‐*A^y^* mice. Four‐week‐old male KK‐*A^y^* mice were divided into four groups. The Control group was fed with AIN93G‐modified high‐fat (3 wt% soybean oil + 17 wt% lard) diet, and the other groups were fed with high‐fat diet, in which 7 wt% of the lard contained in the Control diet was replaced with SCO‐M, SCO‐U, or tuna oil (TO). After the mice had been fed with the experimental diet for 49 days, their serum, liver, and fecal lipid contents, as well as their liver messenger ribonucleic acid expression levels, were evaluated. The SCO‐M and SCO‐U groups were significantly decreased liver cholesterol contents compared to those of the Control and TO groups, partially through the enhancement of the fecal neutral sterol excretions and the tendency to increase the cholesterol 7α‐hydroxylase expression level of the liver. These results indicated that dietary SCO‐M and SCO‐U exhibited cholesterol‐lowering functions in the liver that can help prevent the development of lifestyle‐related diseases.

## Graphical abstract

Our research team has successfully prepared high‐quality scallop oil (SCO), containing high eicosapentaenoic acid (EPA) and phospholipids (PL) from the internal organs of Japanese giant scallop (Patinopecten yessoensis) which is the largest unutilized marine resource in Japan. The SCO diets decreased liver cholesterol contents compared with the Control and tuna oil diets, partially through the enhancement of the fecal neutral sterols excretions and the tendency to increase the cholesterol 7α‐hydroxylase expression level of the liver. These results indicated that dietary SCO exhibited cholesterol‐lowering functions in the liver that can help prevent the development of lifestyle‐related diseases.

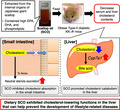

## 1 INTRODUCTION

Cardiovascular diseases (CVD) are one of the leading causes of morbidity and mortality worldwide (World Health Organization, 2017). CVD are mostly caused by atherosclerosis which is formed in the fibrofatty lesions in the artery wall (Libby et al., 2019). The development of fibrofatty lesions requires high cholesterol and triacylglycerol (TAG) contents in the blood (Generoso et al., 2019; Libby et al., 2019). An improvement in dyslipidemia, including high TAG and cholesterol contents in blood, through medicine and food could prevent the development of CVD (Poli et al., 2018). Further, the intake of *n*‐3 polyunsaturated fatty acids (PUFA) including eicosapentaenoic acid (EPA) and docosahexaenoic acid (DHA) reduces the incidence of CVD and mortality through multiple mechanisms, such as the reduction of the serum TAG content, antiplatelet aggregability, and antiarrhythmic effects (Abdelhamid et al., 2018). In addition, the American Heart Association (Kris‐Etherton et al., 2002) recommends the intake of EPA and DHA (1 g/day each), preferably from fish oil, for patients of coronary heart disease. Consequently, EPA and DHA have attracted attention as health‐promoting food materials and the demand for fish oil containing EPA and DHA has increased. Additionally, there are concerns that the rising demand for fish oil will affect marine resources which provide the raw materials for fish oil (Merino et al., 2012).

Predominantly, the species utilized for the production of fish oil are whole fish raw materials, such as the small pelagic fish species (Mylius et al., 2001). The global production of fish oil from whole fish accounts for 74% of the total fish oil production, and the production from by‐products accounts for the other 26% (The Marine Ingredients Organisation, 2016). To ensure a stable population of the Peruvian anchovy (*Engraulis ringens*) which is the main source of fish oil in the country, Peru has limited the fishing of anchovy (Young & Lankester, 2013). Fish oil which produced from by‐products is extensively crucial in overcoming the threats to the marine resources. However, many marine by‐products do not contribute to the production of fish oil. Therefore, there is a strong demand for a new source of *n*‐3 PUFA that could be a supplement to fish oil.

Hokkaido island and Aomori prefecture in northern Japan are the landing areas of scallop (*Patinopecten yessoensis*) with 4.0 × 10^5^–6.0 × 10^5^ tons of scallop landed therein (Kosaka, 2016). In most cases, only the adductor muscles which consist of ~15 wt% of the internal organs are eaten, while other organs, such as the hepatopancreas, gonads, mantles, and gills, are discarded. In 2016, a total of 2.2 × 10^6^ tons of scallop were landed worldwide and the internal organs were discarded (Food & Agriculture Organization of the United Nations, 2020). The hepatopancreas of scallop internal organs contains a large amount of PUFA, especially EPA (Hayashi, 1986, 1988). There have been attempts to prepare oil from the scallop internal organs. However, oil which could satisfy the requirements for utilization as food could not be prepared because of the high levels of toxic compounds, such as diarrheic shellfish poison and cadmium in the hepatopancreas (Matsushima et al., 2018). Therefore, the scallop internal organs have not been employed as a PUFA source. Recently, we developed a method for preparing high‐quality scallop oil (SCO) that satisfied the specifications for utilization, as food, by removing toxic compounds from the scallop internal organs (Okuyama et al., 2016) Additionally, the safety of SCO was confirmed by the studies of single and repeated doses in mice and rats, the bacterial reverse mutation test, and a micronucleus test (Sugimoto et al., 2019; Sugimoto, et al., 2020; Sugimoto, et al., 2020). SCO contains higher EPA than common fish oil (sardine oil); it also contains phospholipids (PL) as well as TAG. However, the health‐promoting function of SCO had not been evaluated. In the present study, we prepared SCOs from the scallop internal organs which were landed in Mutsu (Aomori) and Uchiura (Hokkaido) bays in Japan, and named them SCO‐M and SCO‐U, respectively. The purpose of the present study was to evaluate the effects of dietary SCO‐M and SCO‐U on cholesterol metabolism in obese type‐II diabetic KK‐*A^y^* mice. The KK‐*A^y^* mouse is widely used in the evaluation of the effect of food components on the prevention and improvement of obesity, diabetes, and hyperlipidemia. A KK‐*A^y^* mouse was developed by transferring the *A^y^* gene into a KK mouse, thus inducing obesity and hyperglycemia from the age of 5 weeks (Iwatsuka et al., 1970). EPA and DHA have been reported to demonstrate different health‐promoting functions, such as adiponectin secretion, liver TAG reduction, and prevention of atherogenesis (Guo et al., 2018; Suzuki‐Kemuriyama et al., 2016). Therefore, the effects of SCO were also compared with those of tuna oil (TO), which is rich in DHA.

## 2 MATERIALS AND METHODS

### 2.1 Materials

Soybean oil and lard were purchased from Merck KGaA and Junsei Chemical Co., Ltd., respectively. TO was obtained from Yashima Shoji Co., Ltd.. The ingredients for the experimental diet were purchased from Oriental Yeast Co., Ltd. and Fujifilm Wako Pure Chemical Co.. L‐α‐phosphatidylcholine (PC) from soybean, L‐α‐phosphatidylethanolamine (PE) from egg yolk, and L‐α‐phosphatidyl‐L‐serine (PS) from soybean were purchased from Merck KGaA. All other chemicals (reagent grade) were purchased from Nacalai Tesque, Inc. and Merck KGaA.

### 2.2 Preparation of scallop oils

The scallop internal organs, which were landed from Mutsu bay, consisted of only the hepatopancreas that was collected between April 2017 and May 2017 and was supplied by Sato Chikuro Co.. Another scallop internal organs, including hepatopancreas, gills, mantles, and gonads, landed from Uchiura bay and were collected from September 2017 to October 2017, and were supplied by Yakumo Fishery Cooperative. SCO‐M and SCO‐U were prepared from the scallop internal organs according to the method in the literature (Sugimoto et al., 2019). They were stored in nitrogen headspace gas at −35°C before it was utilized.

### 2.3 Lipid analysis of the experimental oils

The fatty acid (FA) composition was analyzed with a fused‐silica capillary column (Omegawax^®^ 250; Merck KGaA) of a gas chromatography (GC) system (GC‐2014; Shimadzu Co.) after methylation with a boron trifluoride methanol complex solution (Fukunaga et al., 2016). The cholesterol content was analyzed with a fused‐silica capillary column (SH‐Rtx‐5MS; Shimadzu GLC Ltd.) of a GC system after saponification with sodium hydroxide, and 5α‐cholestane was utilized as an internal standard (Kaneda et al., 1980). The PL content was determined by a phosphorus assay (Rouser et al., 1970). Further, the plasmalogen (Pls) contents of SCO‐M and SCO‐U were measured according to the method described in the literature (Williams et al., 1962). The PL class compositions of SCO‐M and SCO‐U were determined with silica gel 60 (Merck KGaA) for thin‐layer chromatography (TLC) employing chloroform/methanol/acetic acid/water (50:40:3:4, v/v/v/v) as the solvent mixture. The PL spots were detected with a 50% (v/v) sulfuric acid solution. Authentic PL standards (ceramide aminoethyl phosphate (CAEP), PC, PE, and PS) were utilized to identify each spot. The CAEP standard of SCO was prepared by the method described in the literature (Sugita et al., 2008). Then, the PL class compositions of SCO‐M and SCO‐U were calculated by spot intensity, employing the JustTLC software (version 4.0.3, Lund, Sweden). The lipid compositions of the experimental oils are given in Table 1.

**Table 1:** Lipid profiles of the experimental oils

	Experimental oils				
	Soybean oil	Lard	SCO‐M	SCO‐U	Tuna oil
Fatty acid composition (wt%)					
C14:0	0.1	1.5	4.8	3.4	2.3
C16:0	9.8	23.0	11.5	15.0	17.0
C16:1*n*‐7	0.1	2.3	11.8	10.0	5.7
C18:0	3.6	12.0	1.7	2.9	3.5
C18:1*n*‐9	24.0	40.0	3.2	5.8	22.0
C18:1*n*‐7	1.9	2.8	4.6	5.9	2.1
C18:2*n*‐6	53.3	15.0	1.2	2.2	0.9
C18:3*n*‐3	6.4	1.4	1.1	1.8	Tr.
C20:4*n*‐6 (AA)	N.D.	N.D.	7.2	4.8	2.6
C20:5*n*‐3 (EPA)	N.D.	N.D.	37.0	25.0	6.2
C22:6*n*‐3 (DHA)	N.D.	N.D.	5.8	14.0	28.0
Others	0.8	2.1	10.1	8.9	10.0
PL (mg/g)	N.D.	N.D.	51.0	166.9	*N*.D.
Pls (mg/g)	‐	‐	0.8	5.8	‐
PL class composition (wt%)					
PC	‐	‐	72.5	65.4	‐
PE	‐	‐	16.5	18.4	‐
PS	‐	‐	4.5	3.1	‐
CAEP	‐	‐	1.9	6.0	‐
Others	‐	‐	7.0	4.6	‐
Cholesterol (mg/g)	N.D.	N.D.	2.7	10.3	1.5

Note

Hyphen (‐) represents the not measured.

Abbreviations: AA, arachidonic acid; and Tr., trace; CAEP, ceramide aminoethyl phosphate; DHA, docosahexaenoic acid; EPA, eicosapentaenoic acid; N.D., not detected; PC, phosphatidylcholine; PE, phosphatidylethanolamine; PL, phospholipids; Pls, plasmalogen; PS, phosphatidylserine; SCO‐M, scallop oil, prepared from scallop internal organs landed from Mutsu bay; SCO‐U, scallop oil, prepared from scallop internal organs landed from Uchiura bay.

### 2.4 Animal diet and care

The experimental protocol followed the “Guide for the Care and Use of Experimental Animals,” issued by the Office of the Japanese Prime Minister, and was reviewed and approved by the Animal Ethics Committee of Kansai University (Approval No. 1819).

Four‐week‐old male KK‐*A^y^* mice were purchased from CLEA Japan, Inc.. The mice were kept in an air‐conditioned room (temperature, 20–22°C; lights on, 08:00–20:00), and they had free access to tap water and the diet. After an acclimatization period of 7 days, the mice were divided into four groups, each consisting of eight mice with similar mean body weight (BW). The mice in the Control group were fed an AIN93G (Reeves et al., 1993) modified high‐fat (3 wt% soybean oil + 17 wt% lard) diet, and the other groups (SCO‐M, SCO‐U, and TO) were fed a high‐fat diet, in which 7 wt% of the lard contained in the Control diet was replaced with SCO‐M, SCO‐U, and TO, respectively. The ingredient and FA compositions of the experimental diets which were analyzed by GC as described above are shown in Tables 2 and 3, respectively. The food intake and BW were measured three times a week during the rearing period.

**Table 2:** Composition of the ingredients of the experimental diets

	Experimental diet			
	Control	SCO‐M	SCO‐U	TO
	g/kg			
Dextrinized corn starch	92.1	92.1	92.1	92.1
Corn starch	277.386	277.386	277.386	277.386
Casein	230	230	230	230
Sucrose	100	100	100	100
Cellulose	50	50	50	50
AIN‐93G mineral mixture	35	35	35	35
AIN‐93 vitamin mixture	10	10	10	10
l‐Cystine	3	3	3	3
Choline bitartrate	2.5	2.5	2.5	2.5
*tert*‐Butylhydroquinone	0.014	0.014	0.014	0.014
Soybean oil	30	30	30	30
SCO‐M		70		
SCO‐U			70	
Tuna oil				70
Lard	170	100	100	100

Abbreviations: SCO‐M, scallop oil, prepared from scallop internal organs landed from Mutsu bay; SCO‐U, scallop oil, prepared from scallop internal organs landed from Uchiura bay; TO, tuna oil.

**Table 3:** Fatty acid composition of the experimental diets

	Experimental diet			
	Control	SCO‐M	SCO‐U	TO
	wt%			
C14:0	1.5	2.5	2.0	1.7
C16:0	22.7	18.8	19.9	20.5
C16:1 *n*‐7	2.3	5.3	4.6	3.3
C18:0	12.4	8.6	9.1	9.2
C18:1*n*‐9	39.6	27.4	27.6	32.9
C18:1*n*‐7	2.8	3.3	3.7	2.6
C18:2*n*‐6	15.1	13.5	14.0	13.0
C18:3*n*‐3	1.4	1.6	1.9	1.4
C20:4*n*‐6 (AA)	N.D.	2.3	1.7	0.9
C20:5*n*‐3 (EPA)	N.D.	11.7	7.6	2.1
C22:6*n*‐3 (DHA)	N.D.	1.8	4.1	8.9
Others	2.2	3.2	3.8	3.5

Abbreviations: AA, arachidonic acid; DHA, docosahexaenoic acid; EPA, eicosapentaenoic acid; *N*.D., not detected; SCO‐M, scallop oil, prepared from scallop internal organs landed from Mutsu bay; SCO‐U, scallop oil, prepared from scallop internal organs landed from Uchiura bay; TO, tuna oil.

After 48 days of administration of experimental diet, the feces of each mouse for 1 day was collected, lyophilized, weighed, and ground in a mill. After the mice had been fed with the experimental diets for 49 days, the mice which were not fasted, were weighed, and thereafter sacrificed after being anesthetized with isoflurane (Fujifilm Wako Pure Chemical Co.) from 9:00 to 12:00. Blood samples were collected without using an anticoagulant, and the serum was obtained by centrifugation for 20 min, at 1,500 × *g*. The liver and white adipose tissue (WAT), including the epididymal WAT, mesenteric WAT, perirenal WAT, and inguinal WAT, were removed rapidly, rinsed with cold saline, and weighed afterward. For the messenger ribonucleic acid (mRNA) expression analysis, a portion of the liver was stored in a RNAlater^®^ solution (Merck KGaA). The serum and organs were frozen in liquid nitrogen and stored at −80°C until analysis.

### 2.5 Lipid analysis of the biological samples

The serum TAG, PL, total cholesterol, high‐density lipoprotein cholesterol (HDL‐C), and non‐high‐density lipoprotein cholesterol (non‐HDL‐C) contents were measured with an Olympus AU5431 automatic analyzer (Olympus Co.) at Japan Medical Laboratory.

The liver total lipid was extracted using the method of Bligh and Dyer (1959). The liver TAG and PL contents were measured by Triglyceride E Test (Fujifilm Wako Pure Chemical Co.) and a phosphorus assay (Rouser et al., 1970). The cholesterol contents in the liver and epididymal WAT were analyzed by GC as described above. The main FA contents of the total lipid were also determined by GC as described above, and utilizing tridecanoic acid (C13:0‐FA), as an internal standard.

The fecal neutral sterol, including the cholesterol and coprostanol, contents were analyzed by GC, as described above (Kaneda et al., 1980). The fecal total bile acid (BA) content was analyzed with the total bile acid test kit (Fujifilm Wako Pure Chemical Co.), following the manufacturer's instructions. The fecal total sterol contents were calculated by adding the neutral sterols and the total BA contents.

### 2.6 Analysis of the liver mRNA expression level

After the liver preserved in RNAlater^®^ solution was crushed with a bead beater‐type homogenizer (MicroSmash MS‐100R, Tomy Seiko Co., Ltd.), the total RNA was isolated and purified, employing the TRIzol^®^ reagent (Thermo Fisher Scientific Inc.), following the manufacturer's protocol. The content and purity of the total RNA were measured at wavelengths of 260 and 280 nm by ultraviolet spectroscopy (UV‐1800, Shimadzu Co.), utilizing Hellma TrayCell^®^ (Hellma GmbH & Co. KG). Thereafter, the complementary deoxyribonucleic acid (cDNA) was synthesized employing the GoScript™ Reverse Transcription System (Promega Co.). The mRNA expression level was measured by the Thermal Cycler Dice^®^ Real‐Time System (Takara Bio Inc.), employing the GoTaq^®^ qPCR Master Mix (Promega Co.). The primer sequences employed for the detection of the adenosine triphosphate (ATP)‐binding cassette (*Abc*) *a1*, *Abcg5*, *Abcg8*, acyl‐coenzyme A cholesterol acyltransferase‐1 (*Acat‐1*), cholesterol 7α‐hydroxylase (*Cyp7a1*), 3‐hydroxy‐3‐methylglutaryl coenzyme A reductase (*Hmgcr*), low‐density lipoprotein receptor (*Ldlr*), scavenger receptor class‐B type‐1 (*Sr‐b1*), sterol regulatory element‐binding factor‐2 (*Srebf‐2*), and glyceraldehyde 3‐phosphate dehydrogenase (*Gapdh*) were as follows: forward: 5′‐AGTTTGTGGCCCTTTTGTGG‐3′ and reverse: 5′‐AAGACCAGGGCAATGCAAAC‐3′, for *Abca1*; forward: 5’‐TGCAGAGCGTTTTTCTG ‐3′ and reverse: 5′‐TGTCATGACTGCCTCTACCTTC‐3′, for *Abcg5*; forward: 5′‐ACGGTGGCAAAGACAAATCC‐3′ and reverse: 5′‐TGGCGTTTTGCTCTGTAAACG‐3′, for *Abcg8*; forward: 5′‐ATTTGCTGATGCTGCCGTAG‐3′ and reverse: 5′‐AGCACAACCACACTGAATGC‐3′, for *Acat‐1*; forward: TGGGCATCTCAAGCAAACAC‐3′ and reverse: 5′‐TCAGAGGCTGCTTTCATTGC‐3′, for *Cyp7a1*; forward: 5′‐TTGGTTTCTGGCGCTTTCAG‐3′ and reverse: 5′‐AACACAGCACGGAAAGAACC‐3′, for *Hmgcr*; forward: 5′‐AATGGGGGCAATCGGAAAAC‐3′ and reverse: 5′‐TGGCACTGAAAATGGCTTCG‐3′, for *Ldlr*; forward: 5′‐TTGGCCTGTTTGTTGGGATG‐3′ and reverse: 5′‐TGCTGAGTCCGTTCCATTTG‐3′, for *Sr‐b1*; forward: 5′‐TGCACCAGAGAGCATTTTGC‐3′ and reverse: 5′‐AGGAACAAAGATGCCACAGC‐3′, for *Srebf‐2*; forward: 5′‐ATGACTCTACCCACGGCAAG‐3′ and reverse: 5′‐TACTCAGCACCAGCATCACC‐3′, for *Gapdh*; which were designed, employing Primer3Plus (http://primer3plus.com/). The results were quantified by a comparative method and expressed as a relative level after normalization to the level of the *Gapdh* expression. This was expressed as the fold change in the mRNA expression that is relative to the Control group.

### 2.7 Statistical analysis

The data were expressed as means ± standard error of the mean (*SEM*). The analysis of variance (ANOVA) and Tukey's multiple comparison tests were employed to evaluate the significant differences, which were set at *p* < .05, among the groups employing the GraphPad Prism software (version 7.0d, GraphPad Software, San Diego, CA, USA).

## 3 RESULTS

### 3.1 Growth parameters and relative organ weights

The growth parameters, including the initial BW, final BW, BW gain, food intake, and food efficiency, which were not significantly different among the groups, and the relative organ weights are given in Table 4. The relative epididymal and mesenteric WAT weights of the SCO‐U group were significantly decreased compared to those in the Control group. There were no significant differences in the relative weights of the liver, perirenal and inguinal WAT throughout the groups.

**Table 4:** Growth parameters and organ weights of the mice, fed with the experimental diets for 7 weeks

	Experimental groups			
	Control	SCO‐M	SCO‐U	TO
Growth parameters				
Initial BW (g)	19.1 ± 0.5	18.9 ± 0.3	19.0 ± 0.3	18.9 ± 0.5
Final BW (g)	42.0 ± 1.1	42.6 ± 1.0	39.8 ± 0.9	41.8 ± 0.8
BW gain (g/day)	0.45 ± 0.02	0.47 ± 0.02	0.42 ± 0.02	0.46 ± 0.01
Food intake (g/day)	4.5 ± 0.1	3.8 ± 0.2	3.8 ± 0.1	4.2 ± 0.2
Food efficiency (g/g)^1^	0.11 ± 0.01	0.12 ± 0.01	0.11 ± 0.01	0.11 ± 0.01
Relative organ weight (g/100 g BW)				
Liver	5.93 ± 0.10	6.35 ± 0.12	6.39 ± 0.20	6.70 ± 0.15
Epididymal WAT	4.28 ± 0.19^b^	3.47 ± 0.15^ab^	2.93 ± 0.28^a^	3.60 ± 0.21^ab^
Mesenteric WAT	3.03 ± 0.06^b^	2.93 ± 0.03^ab^	2.80 ± 0.08^a^	2.69 ± 0.07^a^
Perirenal WAT	2.59 ± 0.33	2.52 ± 0.33	2.17 ± 0.30	2.10 ± 0.15
Inguinal WAT	3.31 ± 0.16	2.74 ± 0.18	2.70 ± 0.20	2.74 ± 0.28

Note

Data represent means ± *SEM* (*n* = 8). Values in the same row not sharing a common superscript are significantly different, at *p* < .05 (Tukey's multiple comparison test).

Abbreviations: BW, body weight; SCO‐M, scallop oil, prepared from scallop internal organs landed from Mutsu bay; SCO‐U, scallop oil, prepared from scallop internal organs landed from Uchiura bay; TO, tuna oil; WAT, white adipose tissue.

^1^ Food efficiency was calculated by dividing BW gain by food intake.

### 3.2 Lipid contents in the biological tissues

The lipid contents of the serum, liver, and epididymal WAT are shown in Table 5. The serum TAG, non‐HDL‐C, and PL contents of the SCO‐M, SCO‐U, and TO groups were significantly decreased, compared to those in the Control group. The serum total cholesterol and HDL‐C contents in the SCO‐M group were significantly lower than those in the Control group. The SCO‐M diet significantly decreased the liver TAG content, compared to the TO diet. The liver cholesterol contents of the SCO‐M and SCO‐U groups were significantly decreased, compared to those of the Control and TO groups. The liver PL and epididymal WAT cholesterol contents were not significantly different among the groups.

**Table 5:** Lipid contents of the biological tissues of mice, fed with the experimental diets for 7 weeks

	Experimental groups			
	Control	SCO‐M	SCO‐U	TO
Serum (mg/dL)				
TAG	555 ± 84^b^	372 ± 57^a^	368 ± 45^a^	256 ± 36^a^
PL	371 ± 22^b^	264 ± 6^a^	303 ± 12^a^	287 ± 15^a^
Total cholesterol	179 ± 11^b^	133 ± 4^a^	156 ± 7^ab^	164 ± 9^ab^
HDL‐C	128 ± 8^b^	97 ± 4^a^	120 ± 5^ab^	126 ± 5^ab^
Non‐HDL‐C	50 ± 4^b^	36 ± 2^a^	35 ± 2^a^	39 ± 3^a^
Liver (mg/g)				
TAG	70.4 ± 4.8^ab^	66.0 ± 4.1^a^	61.1 ± 6.4^a^	88.4 ± 12.2^b^
PL	20.9 ± 0.5	21.0 ± 0.4	24.1 ± 2.5	22.0 ± 0.5
Cholesterol	3.03 ± 0.07^b^	1.80 ± 0.06^a^	1.73 ± 0.05^a^	3.62 ± 0.03^b^
Epididymal WAT (mg/g)				
Cholesterol	0.81 ± 0.09	0.97 ± 0.08	0.86 ± 0.10	0.83 ± 0.14

Note

Data represent means ± *SEM* (*n* = 8). Values in the same row not sharing a common superscript are significantly different, at *p* < .05 (Tukey's multiple comparison test).

Abbreviations: HDL‐C, high‐density lipoprotein cholesterol; Non‐HDL‐C, non‐high‐density lipoprotein cholesterol; PL, phospholipids; SCO‐M, scallop oil, prepared from scallop internal organs landed from Mutsu bay; SCO‐U, scallop oil, prepared from scallop internal organs landed from Uchiura bay; TAG, triacylglycerol; TO, tuna oil; WAT, white adipose tissue.

### 3.3 Main FA contents of the liver

The main FA contents of the liver total lipid are shown in Table 6. The liver EPA contents of the SCO‐M and SCO‐U groups increased significantly, compared with those of the Control group. Additionally, the liver DHA content of the TO group was significantly higher than those of the other groups. The SCO‐M diets significantly increased the liver arachidonic acid (AA), compared to the SCO‐U one. There were no significant differences in the other FA contents.

**Table 6:** Main fatty acid content of the liver of mice, fed with the experimental diets for 7 weeks

	Experimental groups			
	Control	SCO‐M	SCO‐U	TO
	mg/g			
C16:0	175.4 ± 21.2	219.5 ± 32.5	181.2 ± 24.7	253.6 ± 44.0
C16:1*n*‐7	13.5 ± 1.7	21.4 ± 3.4	14.7 ± 2.0	17.0 ± 3.4
C18:0	49.3 ± 5.9	62.4 ± 9.8	45.9 ± 5.0	64.8 ± 8.7
C18:1*n*‐9	290.1 ± 35.1	202.1 ± 32.0	183.3 ± 27.9	352.5 ± 72.4
C18:1*n*‐7	16.5 ± 1.9	15.6 ± 2.5	12.2 ± 1.5	15.8 ± 3.4
C18:2*n*‐6	115.6 ± 13.4	116.3 ± 19.5	103.6 ± 16.1	151.6 ± 24.8
C18:3*n*‐3	4.4 ± 0.5	8.6 ± 1.5	6.7 ± 1.1	7.9 ± 1.1
C20:1*n*‐9	7.9 ± 0.9	5.0 ± 0.8	4.6 ± 0.7	11.3 ± 2.8
C20:4*n*‐6 (AA)	25.9 ± 3.2^ab^	45.1 ± 7.2^b^	17.9 ± 1.6^a^	29.1 ± 4.3^ab^
C20:5*n*‐3 (EPA)	2.4 ± 0.7^a^	52.3 ± 8.9^c^	31.6 ± 4.0^bc^	22.3 ± 2.6^ab^
C22:6*n*‐3 (DHA)	33.9 ± 4.7^a^	74.2 ± 11.1^a^	73.5 ± 9.1^a^	169.6 ± 15.3^b^

Note

Data represent means ± *SEM* (*n* = 8). Values in the same row not sharing a common superscript are significantly different, at *p* < .05 (Tukey's multiple comparison test).

Abbreviations: AA, arachidonic acid; DHA, docosahexaenoic acid; EPA, eicosapentaenoic acid; SCO‐M, scallop oil, prepared from scallop internal organs landed from Mutsu bay; SCO‐U, scallop oil, prepared from scallop internal organs landed from Uchiura bay; TO, tuna oil.

### 3.4 Fecal sterol excretions

The fecal sterol excretions are shown in Table 7. Fecal neutral sterols including cholesterol and coprostanol, and excretions in the SCO‐M and SCO‐U groups were significantly increased compared with that in the Control and TO groups. The SCO‐M and SCO‐U groups were significantly higher in fecal total sterol excretions than the Control group. The SCO‐U diet increased the fecal neutral sterols, including cholesterol and coprostanol, and the total sterol excretions than the SCO‐M diet. Moreover, there was no significant difference in the total fecal BA excretion among the groups.

**Table 7:** Fecal sterol excretion in mice, fed with the experimental diets for 7 weeks

	Experimental groups			
	Control	SCO‐M	SCO‐U	TO
	mg/day			
Neutral sterols^1^	0.43 ± 0.16^a^	1.41 ± 0.17^b^	2.41 ± 0.25^c^	0.62 ± 0.06^a^
Cholesterol	0.05 ± 0.01^a^	0.58 ± 0.05^b^	1.20 ± 0.12^c^	0.05 ± 0.02^a^
Coprostanol	0.47 ± 0.16^a^	1.99 ± 0.20^b^	3.61 ± 0.36^c^	0.66 ± 0.06^a^
Total BA	1.01 ± 0.22	1.21 ± 0.25	1.51 ± 0.19	1.52 ± 0.23
Total sterols^2^	1.48 ± 0.24^a^	3.20 ± 0.43^b^	5.12 ± 0.32^c^	2.19 ± 0.26^ab^

Note

Data represent means ± *SEM* (*n* = 8). Values in the same row not sharing a common superscript are significantly different, at *p* < .05 (Tukey's multiple comparison test).

Abbreviations: BA, bile acids; SCO‐M, scallop oil, prepared from scallop internal organs landed from Mutsu bay; SCO‐U, scallop oil, prepared from scallop internal organs landed from Uchiura bay; TO, tuna oil.

^1^ Neutral sterols are the sum of cholesterol and coprostanol.

^2^ Total sterols are the sum of the neutral sterols and total BA.

### 3.5 Liver mRNA expression level related to cholesterol metabolism

**FIGURE 1:**
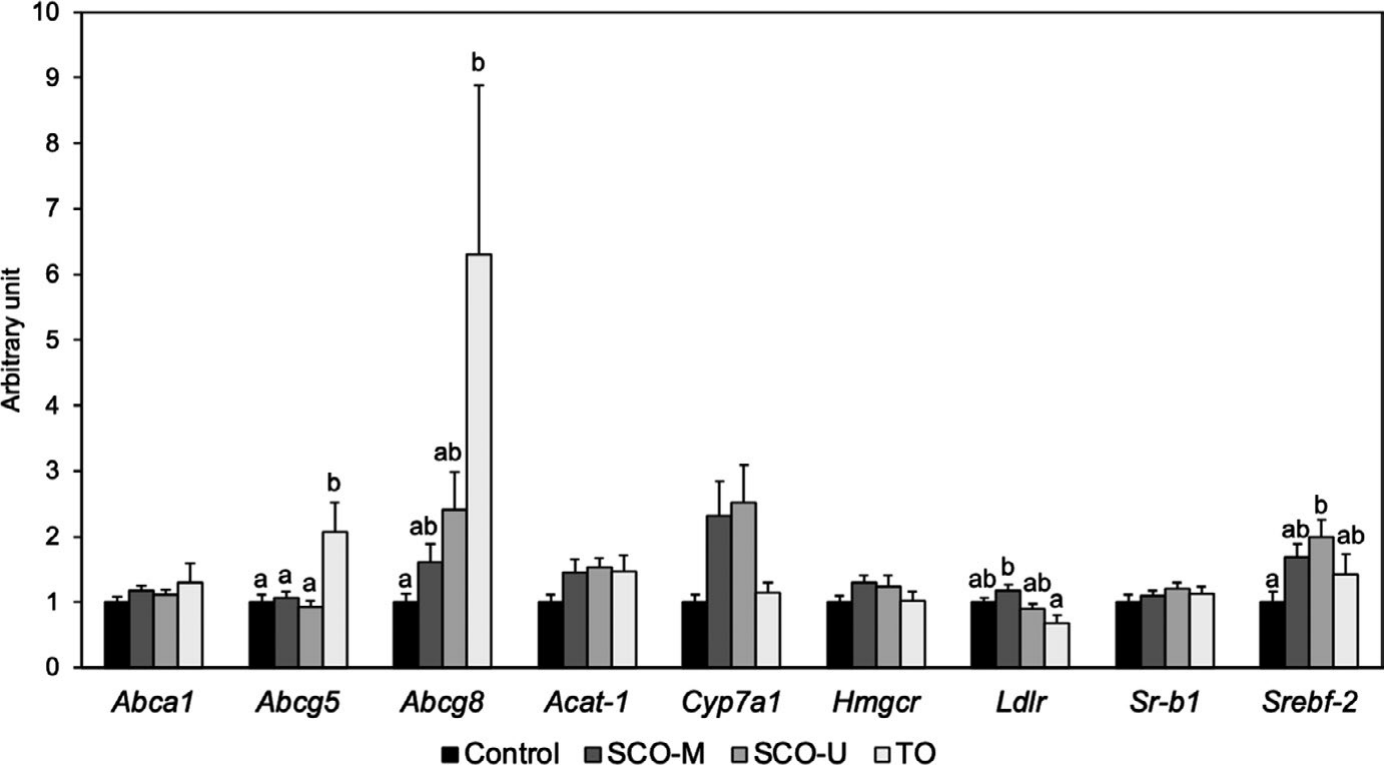
Liver mRNA expression levels, related to cholesterol metabolism in the mice, fed with the experimental diets for 7 weeks. Data represent means ± *SEM* (*n* = 8). Values in the same row not sharing a common superscript are significantly different, at *p* < .05 (Tukey's multiple comparison test). Results were quantified, employing a comparative method, and were expressed as a relative level, after normalization, to the level of the *Gapdh* expression and expressed as the fold change in the mRNA expression, relative to the Control group (set at 1.00). *Abca1*; ATP‐binding cassette A1, *Abcg5*; ATP‐binding cassette G5, *Abcg8;* ATP‐binding cassette G8, *Acat‐1*; acyl‐coenzyme A: cholesterol acyltransferase‐1, *Cyp7a1*; cholesterol 7α‐hydroxylase, *Gapdh*; glyceraldehyde 3‐phosphate dehydrogenase, *Hmgcr*; 3‐hydroxy‐3‐methylglutaryl coenzyme A reductase, *Ldlr*; low‐density lipoprotein receptor, *Sr‐b1*; scavenger receptor class‐B type‐1, *Srebf‐2*; sterol regulatory element‐binding factor‐2; SCO‐M, scallop oil, prepared from scallop internal organs landed from Mutsu bay; SCO‐U, scallop oil, prepared from scallop internal organs landed from Uchiura bay; TO, tuna oil

The liver mRNA expression levels, related to cholesterol metabolism, are shown in Figure 1. The liver *Srebf‐2* expression level in the SCO‐U group significantly increased, compared to those in the Control group. The SCO‐M and SCO‐U groups tended to be higher in the *Cyp7a1* expression level than the Control group, *p* = .13 and .06, respectively. The TO group significantly increased in the *Abcg5* and *Abcg8* expression levels, compared to the Control group. There were also no significant differences in the *Abca1*, *Acat‐1*, *Hmgcr*, *Ldlr*, and *Sr‐b1* expression levels among the groups.

## 4 DISCUSSION

SCO‐M and SCO‐U were characterized by the presence of 37.0 and 25.0 wt% of EPA and 51.0 and 166.9 mg/g of PL, respectively, whereas TO contained only 6.2 wt% of EPA and did not contain PL (Table 1). The high EPA content of SCO‐M was attributed to the high EPA content of the hepatopancreas of the scallop, obtained in April–May (Hayashi, 1986). Moreover, since the hepatopancreas were stored in TAG (Hayashi, 1988), the low PL content of SCO‐M which was prepared from the only hepatopancreas, was due to the high amount of TAG, collected from the hepatopancreas. Additionally, SCO‐M and SCO‐U were contained in Pls, which is a subclass of PL, possessing a vinyl ether bond with FA at the *sn*‐1 position of glycerol and CAEP which possessed a C–P bond, formed by a phosphorus atom of 2‐aminoethylphosphonate (Table 1). Further, the various physiological activities of Pls and CAEP have been reported (Che et al., 2018; Ifuku et al., 2012; Komatsu et al., 2013). For example, Ifuku et al. (2012) reported that Pls intake demonstrated antineuroinflammatory and antiamyloidogenic effects on the brains of the mice with lipopolysaccharide‐induced inflammation, and Che et al. (2018) also suggested that the intake of EPA‐enriched Pls improved the learning and memory functions of senescence‐accelerated mouse prone 8. Moreover, Komatsu et al. (2013) reported that CAEP exerted a protective effect against microcystin‐LR induced liver injury. Therefore, SCOs were expected to exhibit better health‐promoting functions than typical fish oils because it contained PL including Pls and CAEP, which were not detectable in typical fish oil. The ratio of the Pls contents of SCO‐M and SCO‐U was higher than that of the PL contents in both oils (Table 1). Further, the CAEP compositions of SCO‐M and SCO‐U were also different. The differences in the Pls contents and CAEP compositions of SCO‐M and SCO‐U were considered to be caused by the differences in the parts of the internal organs, which were employed for the preparation of the oils or the landing period. Thus, further experiments are required to clarify these points.

The SCO‐U diet significantly decreased the relative epididymal and mesenteric WAT weights, compared to the Control group (Table 4). A previous study reported that the intake of EPA and DHA decreased the WAT weights through the enhancement of mitochondrial biogenesis and the induction of β‐oxidation in WAT of mice (Flachs et al., 2005). Additionally, a previous study demonstrated that the intake of EPA and DHA reduced the total WAT weight, compared to the intake of corn oil, in a mice‐fed high‐fat diet (Soni et al., 2019). The decrease in the relative epididymal and mesenteric WAT weights in the SCO‐U group could be due to dietary EPA and DHA in SCO‐U. However, the food intake of each group was not equal, and the decrease in the relative epididymal and mesenteric WAT weights of the SCO‐U group could be slightly affected by the differences in the food intake among the groups. Therefore, a pair‐feeding study was required to determine whether SCO‐U intake could reduce the epididymal and mesenteric WAT weights.

The main FA contents of the total liver lipids were revealed by the FA compositions of the experimental oils (Tables 3 and 6). The SCO‐M group was significantly higher in liver AA content than the SCO‐U group (Table 6). SCO‐M contains 7.2 wt% AA of the total FA composition, whereas SCO‐U contains 4.8 wt% AA of it. The high liver AA content of the SCO‐M group was due to the difference between the AA compositions of SCO‐M and SCO‐U. Conversely, compared to the Control group, the liver EPA contents of the SCO‐M and SCO‐U groups significantly increased, and the liver DHA content of the TO group significantly increased (Table 6). The liver FA metabolism was mainly regulated by transcriptional factors, including the sterol regulatory element‐binding protein (SREBP)‐1 and SREBP‐2 and nuclear receptors, including the liver X receptor (LXR) and peroxisome proliferator‐activated receptor alpha (PPARα) (Horton et al., 1998; Schoonjans et al., 1996; Shimano et al., 1996). EPA and DHA suppressed the synthesis of liver FA through SREBP‐1c expression because it competed with the LXR ligand in the activation of the ligand‐binding domain of LXR (Yoshikawa et al., 2002). Moreover, EPA and DHA are natural ligands of PPARα, which regulated the stimulated β‐oxidation of FA (Grygiel‐Górniak, 2014). From this information, EPA and DHA intake decreased the serum and liver lipid contents because of the suppression of FA synthesis and the enhancement of FA oxidation through the regulations of LXR and PPARα (Ide et al., 2000; Kim et al., 1999). Here, the serum TAG contents of the SCO‐M, SCO‐U, and TO groups significantly decreased, compared to those of the Control group (Table 6). These results suggested that the intake of EPA and DHA, contained in SCO‐M, SCO‐U, and TO, could lower the serum TAG content partly through the regulation of the SREBP‐1c and PPARα expressions. Additionally, the serum TAG content of the TO group decreased by 30% and 31% compared to those of the SCO‐M and SCO‐U groups, respectively (Table 6). Contrarily, the liver TAG content of the TO group significantly increased, compared to that of the SCO‐M and SCO‐U groups (Table 6). A previous study suggested that the dietary DHA bound *sn*‐2 position of TAG decreased the very‐low‐density lipoprotein (VLDL) content of serum by 37%, compared to the dietary EPA bound *sn*‐2 position of TAG in mice (Yoshinaga et al., 2015). Therefore, dietary TO could decrease the serum TAG content than the dietary SCO‐M and SCO‐U by suppressing VLDL excretion from the liver into the blood.

Compared to the Control and TO groups, the SCO‐M and SCO‐U groups significantly decreased the cholesterol contents of the liver (Table 6). Several mechanisms could explain the cholesterol‐lowering function of the dietary oils. One possibility is enhanced fecal sterol excretions (Hosomi et al., 2012). The SCO‐M and SCO‐U diets significantly increased the fecal neutral sterols, namely cholesterol and coprostanol excretions than the Control and TO groups (Table 7). Further, the fecal total sterol excretions of the SCO‐U group were higher than that of the Control and TO groups. The glycerophospholipid (GPL) intake is generally well known to inhibit cholesterol absorption in the small intestine (Cohn et al., 2010). The excretions of the fecal neutral sterols, including cholesterol and coprostanol, were significantly higher in the mice, fed with the SCO‐U diet containing high PL content, than in the mice, fed with the SCO‐M diet, which possessed low PL content. The previous study reported that the GPL intake inhibited cholesterol absorption in the small intestine by inhibiting the hydrolysis of the micellar PL by phospholipase A2 (Lee et al., 2019). Additionally, PC and PE, which were of GPL class, decreased the cholesterol contents of the serum through the inhibition of cholesterol absorption in the small intestine (Imaizumi et al., 1983; Jiang et al., 2001). Conversely, sphingomyelin (SM), a kind of sphingolipid, also inhibited cholesterol absorption through hydrogen bonding with the hydroxyl group of cholesterol (Noh & Koo, 2004; Ohvo‐Rekilä et al., 2002). Further, ceramide and sphingoid bases, which are products of SM digestion, also inhibited the intestinal absorption of cholesterol because of the interactions between the hydroxyl group of cholesterol and the carboxylic acid group of FA (Garmy et al., 2005). Although no study has reported the inhibition of cholesterol absorption by CAEP intake, CAEP possesses the potential to inhibit cholesterol absorption, as SM, because CAEP had been hydrolyzed into free sphingoid bases during the digestion process (Tomonaga et al., 2017). In the present study, the PL class compositions of SCO‐M and SCO‐U consisted of 72.5 and 65.4 wt% PC, 16.5 and 18.4 wt% PE, and 1.9 and 6.0 wt% CAEP, respectively (Table 1). Therefore, the enhancement of the fecal neutral sterol excretions by the intake of SCO‐M and SCO‐U was influenced by the inhibition of cholesterol absorption by PC, PE, and CAEP.

However, there was a limitation in the fecal sterol excretions. Since SCO‐M and SCO‐U contained cholesterol, the fecal sterol excretions in the SCO‐M and SCO‐U groups could be influenced by cholesterol. The average dietary cholesterol contents in the SCO‐M and SCO‐U groups are 0.76 and 2.7 mg/day, respectively. Therefore, the possibility that dietary cholesterol in the SCO‐M and SCO‐U groups could affect the fecal sterol excretions could not be neglected. Although SCO‐M and SCO‐U contained cholesterol, their intake reduced the cholesterol contents of the liver. For a more accurate assessment of the effects of SCO‐M and SCO‐U intake on inhibiting cholesterol absorption, further experiments should be conducted on diets containing the same cholesterol content.

The other possibility of the cholesterol‐lowering mechanism could be related to the metabolic balance among the biosynthesis, catabolism, incorporation, and excretion in sterol metabolism. Therefore, the liver mRNA expression levels related to cholesterol metabolism were evaluated. The liver *Abca1*, which formed a pre‐β high‐density lipoprotein by adding PL to apolipoprotein A‐I, *Abcg5/8*, excreted cholesterol to bile, *Acat‐1*, esterified cholesterol, and *Ldlr* and *Sr‐b1*, which are cholesterol carriers from the blood, ensured that there were no significant differences in the expression levels of the SCO‐M and SCO‐U groups were, compared to those of the Control group (Figure 1). Conversely, the liver *Abcg5/8* expression levels of the TO group were significantly increased, compared to those of the Control group. Our previous study demonstrated that the dietary TO significantly increased the liver *Abcg5/8* expression levels compared to the dietary menhaden oil which is rich in EPA (Hosomi et al., 2019), in rats. Therefore, the dietary DHA was expected to increase cholesterol excretion from the liver to bile by increasing the liver *Abcg5/8* expression levels. *Hmgcr* is the rate‐limiting enzyme for the synthesis of cholesterol, which is regulated by SREBP‐2. SCO‐U intake significantly increased the liver *Serbf‐2* expression level, compared to the intake of the Control group, although the liver *Hmgcr* expression level was not changed by the intake of SCO‐U. With the liver *Cyp7a1*, the rate‐limiting enzyme in the classical BA biosynthetic pathway, the expression levels in the SCO‐M and SCO‐U groups tended to increase, compared to those of the Control group (*p* = .13 and .06, respectively). This phenomenon might have contributed to decreasing the cholesterol contents of the liver of the SCO‐M and SCO‐U groups. Our previous study indicated that the dietary GPL containing PUFA which mainly consisted of PC and PE increased the liver *Cyp7a1* expression level and decreased the liver cholesterol content, compared to dietary TAG containing PUFA, in rats (Hosomi et al., 2012). Additionally, Ding et al. (2020) suggested that the intake of EPA‐enriched Pls dramatically decreased liver cholesterol content through the increased liver *Cyp7a1* expression level. Therefore, in this study, the tendency of increasing the liver *Cyp7a1* expression levels in the SCO‐M and SCO‐U groups may depend on dietary GPL, such as PC, PE, and Pls, contained in SCO‐M and SCO‐U.

## 5 CONCLUSION

This study evaluated the effects of dietary SCO‐M and SCO‐U on cholesterol metabolism in obese type‐II diabetic KK‐*A^y^* mice. The intake of SCO‐M and SCO‐U decreased the cholesterol contents of the liver, which was not observed in the TO intake. The reductions in the liver cholesterol content because of SCO‐M and SCO‐U intake were mediated by the enhancement of total fecal sterol excretions and the tendency to increase the liver *Cyp7a1* expression level. Therefore, SCO, including PUFA and PL, is expected to be a food material with health‐promoting properties, which could be an alternative to fish, for the production of oil.

## CONFLICT OF INTEREST

All authors declare no conflicts of interest.
